# A71 SUBGROUP ANALYSIS OF CANADIAN PARTICIPANTS IN PUNCH CD3-OLS: A PHASE 3 OPEN-LABEL CLINICALSTUDY TO EVALUATE THE SAFETY AND EFFICACY OF FECAL MICROBIOTA, LIVE-JSLM, IN ADULTS WITH RECURRENT *CLOSTRIDIOIDES DIFFICILE* INFECTION

**DOI:** 10.1093/jcag/gwae059.071

**Published:** 2025-02-10

**Authors:** T S Steiner, T Louie, D Kao, D Smyth, B Guthmueller, T Jason, S N Elliott, S Srivastava, C Lee

**Affiliations:** University of British Columbia, Vancouver, BC, Canada; University of Calgary, Calgary, AB, Canada; University of Alberta, Edmonton, AB, Canada; Dalhousie University, Moncton, NS, Canada; Ferring Pharmaceuticals Inc, Parsippany, NJ; Ferring Inc., North York, ON, Canada; Ferring Inc., North York, ON, Canada; Ferring Pharmaceuticals A/S, Copenhagen, Denmark; University of Victoria, Victoria, BC, Canada

## Abstract

**Background:**

Fecal microbiota, live-jslm (RBL) is the first United States (US) Food and Drug Administration-approved, single-dose, microbiota-based live biotherapeutic product to prevent recurrent *Clostridioides difficile* infection (rCDI) in adults following standard-of-care (SOC) antibiotics. In a prospective phase 3, open-label study (PUNCH CD3-OLS; NCT03931941) conducted in the US and Canada, RBL demonstrated a 73.8% treatment success rate at 8 weeks and a 91.0% sustained clinical response rate through 6 months.

**Aims:**

To evaluate the safety and efficacy of RBL in Canadian participants in PUNCH CD3-OLS.

**Methods:**

PUNCH CD3-OLS participants were aged ≥18 years with documented rCDI and confirmed use of SOC antibiotics. Participants with comorbidities including inflammatory bowel disease (IBD), irritable bowel syndrome (IBS), and immunocompromising conditions due to disease and/or medications were permitted to enroll. A single dose of RBL was rectally administered within 24–72h of SOC antibiotic completion. The primary endpoint was the number of participants with RBL- or administration-related treatment-emergent adverse events (TEAEs). Secondary endpoints included treatment success at 8 weeks and sustained clinical response at 6 months after RBL administration.

**Results:**

Of the 697 participants who received RBL, 117 were Canadian. Participants in Canada were mostly White (91.5%) and female (70.1%) with a mean (SD) age of 61.3 years (17.38). Twenty participants (17.1%) were hospitalized for their qualifying episode. Participants’ medical histories included IBD (unspecified, [1.7%, n=2]; Crohn’s disease, [6.0%, n=7]; ulcerative colitis, [5.1%, n=6]), IBS (6.8%, n=8), and mild-to-moderate immunocompromising conditions (7.7 %, n=9). TEAEs through 8 weeks after RBL administration were reported by 59.8% of participants; most were gastrointestinal (48.7%, n=57) and mild-to-moderate in severity. Serious TEAEs were reported by 5.1% of participants within 8 weeks of RBL administration. At 8 weeks, 75.2% of participants (88/117) had treatment success; of those, 90.9% (80/88) had a sustained clinical response at 6 months. Demographics, safety, and efficacy outcomes were similar for participants in the US.

**Conclusions:**

RBL was safe and efficacious for preventing rCDI among Canadian participants from PUNCH CD3-OLS, the largest study of a live microbiota-based therapy conducted to date.

**Funding Agencies:**

NRCFerring Pharmaceuticals

